# German Correspondence

**Published:** 1870-01

**Authors:** 


### GERMAN CORRESPONDENCE.

A very young lady writes to the editor as follows, from Heidelberg, Germany, under date of July 20, 1869:

Perhaps by the time this reaches you, you will be at the Bay of Fundy. For father's sake, I am heartily glad, as it has been, if I am not very much mistaken, the dream of his life to see that place. When he reaches it, I think one of the wrinkles will be removed from his forehead, if not the whole of them.

I have been waiting this long time in the hope of sending you something in my next, and thought at last that I had three facts certain to state to you, but that hope was nipped in the bud; wait, and I will explain.

PHANTOM NO. 1.

I heard that I was going to be invited to a party; great excitement prevails; head quite in a whirl about gloves, slippers, dress, possible conquests, and a drive home in the evening with some new exquisite. Alas! heard that said party was composed of foreigners only. I declined, and became disconsolate.

PHANTOM NO. 2.

Case of wounded pride. Am invited to sing in the choir. Do all the people who look at me so carelessly on the street know that I sing in the choir? I rush tempestuously to church, rise with expanded lungs and lofty mien; where is the music? minister turns pale, the congregation in general haste to the rescue; afterward learned I was invited to take the place of the best voice (absent in Switzerland), and must warble with the mediocrity a second time.

PHANTOM NO. 3.

Announced that there would be a ball at No. 58, but found upon investigation that there were certain antipathies among the guests intended to be invited. It was not to have been a ball really, only a few friends were to be invited; but the plan fell through; so you see I have nothing to tell but "that which might have been."

It is too delightful to see people from America, let alone acquaintances; we have met two already this summer, Mr. H. and others, and felt almost inclined to embrace them.

HOTEL HERMAN.

July 31.—You see by this that I have left Heidelberg. Cronstadt is a watering-place, about two miles out of Stuttgart. The hotel is an immense one, and was so crowded when I came that I was obliged to take a small room on the fourth floor, but tò-day have changed to one much more pleasant. There were two hundred people at dinner last Sunday. I enjoy it immensely, and I do wish you would hurry back, so we could stay at hotels all the time.

There is a band of music two or three times a week, dancing, croquet, etc. There are several American families here, and it is so nice to be meeting new people all the time; there is one lady here who has been in Rome fourteen years. Mrs. H. knows so many people that it is always pleasant.

Just before I left Heidelberg I was at the Castle with Rev. Mr. C., and who should I meet but B. from New York, who is going up the Nile this winter, having just left Paris on the way to Baden Baden.

This is one of the least expensive hotels we have been at, and it is certainly very comfortable. I have a good-sized front room, with bureau, settee and easy chair, besides the furniture one always finds in a hotel room. It is very expensive traveling in Switzerland and Italy.

August 5.—Mrs. H. came yesterday to take a drive to Stuttgart. A friend came here yesterday who had taken rooms at the Fifth Avenue Hotel in New York after selling their house, and remained there two or three months at twenty-five dollars a day, and that was less than they could keep house for; no wonder they consider prices here so moderate.

You seem to be well known to all the Americans here through your books; one lady says she and her sister became very much taken with a piece of advice given by you to mothers: "When children are hurt, do not take them up and pity them, but direct them." They have tried it, and found it worked splendidly. Another lady, the one who has been in Rome so long, seems very much inclined to quarrel with you, because you forbid drinking at meal times; but she is the healthiest-looking specimen I have seen for a long time, and I guess she eats and drinks what she pleases.

LEARNING DUTCH.

Alice is forgetting entirely the idiomatic construction of the English language. She often makes such remarks as the following: "I have got my hair in a knot; you know the *fault* of it." "The joke is *tired* out." She remains true in one respect—her rights are, as formerly, well asserted by herself; no one will ever cheat her.

Bob's whiskers are at length arrived, but the poor child is so afraid of exciting the risibles of some barber, he takes the business of shaving into his own hands—scissors and hand-glass are the instruments. Still I am constantly afraid I shall have to be the bearer of some sad message, that he is minus a cheek, a half lip, or something. This new article in question bears some resemblance to tow.

